# Off-resonant pulmonary vein imaging

**DOI:** 10.1186/1532-429X-11-S1-P185

**Published:** 2009-01-28

**Authors:** Peng Hu, Dana C Peters, Christian Stoeck, Kraig V Kissinger, Beth Goddu, Lois Goepfert, Warren J Manning, Reza Nezafat

**Affiliations:** Beth Israel Deaconess Medical Center, Boston, MA USA

**Keywords:** Pulmonary Vein, Pulmonary Vein Isolation, Inferior Pulmonary Vein, Gradient Recall Echo, SSFP Sequence

## Introduction

Non-contrast enhanced pulmonary vein (PV) MRA is a potential alternative to contrast enhanced methods for pre-procedural planning and post-procedural evaluation of pulmonary vein isolation (PVI) in treatment of atrial fibrillation (AF). In 2007, 12% of the AF patients referred to pulmonary MRA in our center have impaired renal function. A major problem with non-contrast PV imaging is lack of contrast between PV and other great vessels (e.g. pulmonary artery) and cardiac chambers. Because the PV is in close proximity to the lungs, the PV blood could potentially experience significant off-resonance due to susceptibility effects. This off-resonance could be used as a source of contrast to differentiate PV from other anatomical features [1].

## Purpose

We sought to investigate variations of off-resonance frequency through the cardiac cycle for each PV branch and to investigate the use of off-resonance in PV SSFP imaging.

## Methods

PV images were acquired on healthy volunteers in this study. We used a dual echo sequence based on gradient recalled echo (GRE) to measure the field map of the PV and the left atrium in cine mode. The off-resonance of a PV branch was measured by defining a region of interest (ROI) in the proximal PV of the generated field maps and calculating the mean off-resonance frequency in the ROI. A 3D ECG gated free-breathing SSFP sequence with a navigator echo, which has been used in coronary imaging, was adapted to PV imaging. Balanced SSFP has a well-known signal profile modulated by frequency shift. By applying a linearly increasing radiofrequency (RF) excitation phase from TR to TR, we shift the frequency response of SSFP signal so that the off-resonant PV blood signal is enhanced.

## Results

**Figure 1:**
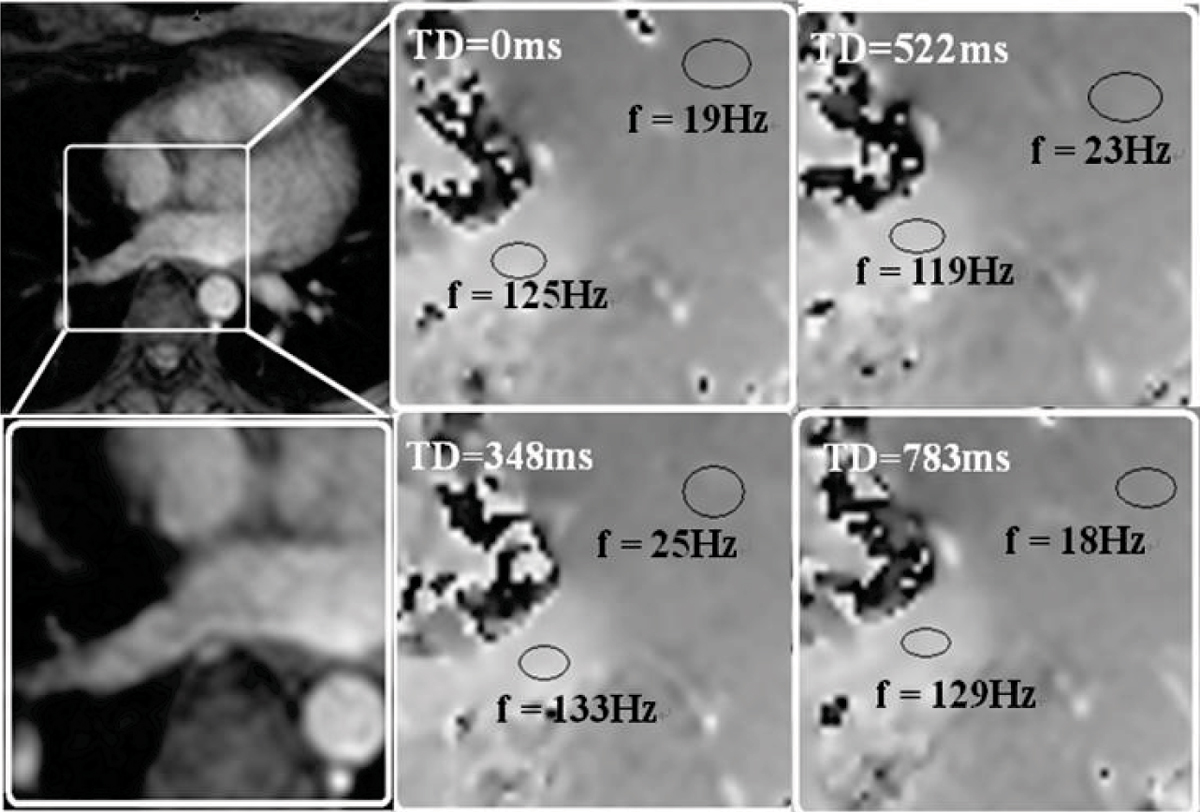
**An example of right inferior PV frequency shift in a healthy subject through the cardiac cycle**. PV images *(left columns)* and the corresponding off-resonance map *(middle and right columns)* at the same imaging plane are shown. The off-resonance at proximal PV and atrial blood are measured. TD = ECG trigger delay.

**Figure 2:**
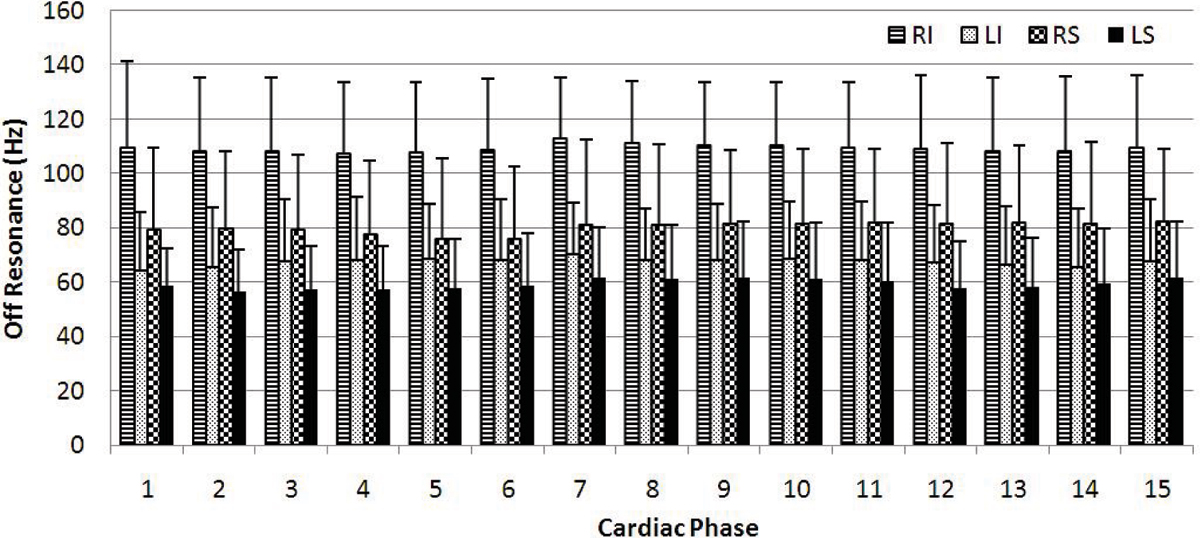
**Variaions of PV off-resonance frequencies between subjects, PV branches and cardiac phase**. RI = right inferior; LI = left inferior; RS = right superior; LS = left superior.

**Figure 3:**
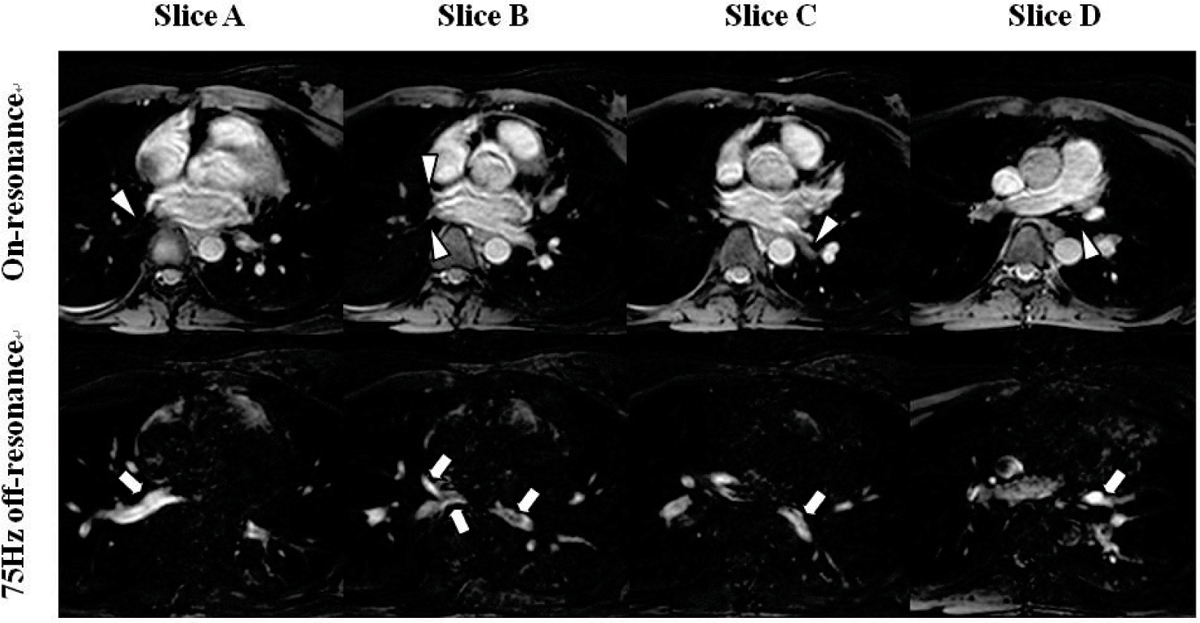
**Example images from a healthy subject using on-resonance (** ***top*** **) and 75 Hz off-resonance (** ***bottom*** **) SSFP sequences**. Slices A, B, C and D are part of a 3D data set. PV branches are highlighted in off-resonance SSFP (*arrows*) whereas they are suppressed in on-resonance SSFP (*arrow heads)*.

Figure 1 shows an example of off-resonance measurements in the right inferior PV from a healthy adult subject through the cardiac cycle. Figure 2 shows the variations of off-resonance between different PV branches and different time in the cardiac cycle based on data from a cohort of 6 healthy subjects. On all six subjects, we observed the largest frequency shift in the right inferior PV among all branches. The mean frequency shifts for all PV branches stay within a narrow range of 10 Hz through the cardiac cycle. Figure 3 shows an example to use the frequency shift as a source of contrast for the PV's. Signal enhancement can be observed in all four major PV branches.

## Conclusion

PV blood exhibits a mean off-resonance of 58~113 Hz, with the right inferior PV having the largest frequency shift. We observed minimal RR variation in frequency off-set. By shifting the frequency response of SSFP sequence, we enhance the PV blood signal compared to on-resonance conditions. A combination of both on- and off-resonance SSFP acquisitions is a potential method to obtain coverage for both left atrium and PV.
